# Correlation of IgG autoantibodies against acetylcholine receptors and desmogleins in patients with pemphigus treated with steroid sparing agents or rituximab

**DOI:** 10.1371/journal.pone.0233957

**Published:** 2020-06-18

**Authors:** Sravya M. Bhatia, Robert D. Streilein, Russell P. Hall

**Affiliations:** 1 Duke University School of Medicine, Durham, North Carolina, United States of America; 2 Department of Dermatology, Duke University School of Medicine, Durham, North Carolina, United States of America; University of Nebraska-Lincoln, UNITED STATES

## Abstract

**Introduction:**

Autoantibodies (autoAbs) against desmoglein-1 (DSG1) and desmoglein-3 (DSG3) have conventionally been studied and well accepted in the pathogenesis of pemphigus vulgaris (PV) and foliaceus (PF). Recent studies have suggested that non-DSG autoAbs may contribute to the pathogenesis of pemphigus, including autoAbs directed at acetylcholine receptors (AChR) and thyroid peroxidase (TPO). The purpose of this study is to retrospectively analyze PV and PF patient sera to better understand the relationship between anti-AChR and -TPO Abs to disease activity and DSG reactivity between patients treated with prednisone and steroid sparing agents (SSA; n = 22) or prednisone and rituximab (n = 21).

**Methods:**

Patients were evaluated at 2 time points, T1 and T2, for disease activity using the Pemphigus Disease Area Index (PDAI), and sera were tested for the presence of TPO, DSG1, DSG3, muscarinic (M3) and nicotinic (n) AChR IgG autoAbs, as well as antibodies against Varicella Zoster Virus (VZV) by ELISA.

**Results:**

Disease activity significantly decreased in patients from T1 to T2 (p < .0001). A significant difference was seen in IgG anti-DSG1 (p < .0001) and anti-DSG3 (p = .0049) levels when T1 was compared to T2 in both treatment groups. A significant increase was found between pemphigus patients and normal subjects with nAChR (p < .0001) at T1 but not with m3AChR, TPO or VZV Abs. No significant difference was seen between T1 and T2 values in patients with pemphigus for the non–desmoglein Abs TPO (p = .7559), M3AChR (p = .9003), nAChR (p = .5143) or VZV (p = .2454). These findings demonstrate that although an increase in IgG anti-nAChR autoAbs was found in PV and PF subjects, these Abs did not decrease with treatment. No other non-DSG Abs were increased or significantly changed over time in patients with pemphigus. This suggests that anti -AChR and -TPO Abs may not play a direct role in the pathogenesis of most patients with pemphigus, but does not rule out a role for non-DSG auto antibodies in distinct subsets of pemphigus patient.

## Introduction

Pemphigus is a group of severe, chronic organ-specific autoimmune blistering diseases characterized by blistering and erosions of the skin and mucous membranes. [1, 2] Desmogleins are desmosomal adhesion molecules, and they are required for keratinocyte cell-to-cell adhesion. [3] The role of autoantibodies (autoAbs) reactive against these intercellular adhesion proteins, desmoglein 1 and 3 (DSG1, DSG3), in the pathogenesis of pemphigus has been well accepted and studied for decades. [4–7] Passive transfer studies using antibodies (Abs) directed against DSG1 and DSG3, as well as studies with DSG3 knockout mice, have shown a clinical phenotype similar to that seen in patients with pemphigus. [8, 9]

Several studies have shown, however, that anti-DSG Ab levels may not correlate with level of disease activity in all patients. Some patients with highly active disease have no measurable DSG Abs and vice versa. [10, 11] This occurrence has thus led to further investigation into the pathogenesis of pemphigus to elucidate the potential differences amongst patients. Recent studies have suggested that another subset of non-DSG autoAbs may contribute to the pathogenesis of pemphigus via a more synergistic, or alternative, multifactorial model of autoAbs coordinating to produce the variations of pemphigus seen in patients. [12–18] Two autoAbs of interest are anti-thyroid peroxidase (anti-TPO) and both muscarinic and nicotinic subtypes of anti-acetylcholine receptor (anti-AChR) IgG autoAbs. [17, 19, 20] Both anti-TPO and anti-AChR have been studied in patients with pemphigus, and some studies suggest a possible correlation of autoAb titers with disease activity, suggesting a role in the pathogenesis of pemphigus. [20–25] Alternatively, it is also possible that these Abs may not be related directly to the pathogenesis of the clinical findings of pemphigus.

Treatment of pemphigus has historically consisted of high doses of corticosteroids and immunosuppressants to reduce overall immune response. [26] However, pemphigus patients often relapse with this standard therapy necessitating long-term steroid use. Patients, thus, often suffer from the severe side effects associated with prolonged steroid use, such as diabetes, hypertension, and infections. Recent studies have shown that treatment with rituximab, a chimeric murine-human monoclonal Ab against CD20 antigen of B cells, depletes peripheral blood B cells and alters the repopulation of B cells when compared to conventional corticosteroid treatment. [27, 28] Furthermore, treatment of pemphigus with rituximab decreases circulating autoAbs against DSG1 and DSG3 without normally impacting Abs directed against existing antigens (e.g., tetanus toxoid or Varicella Zoster Virus). This effect has been suggested to be the results of a decrease in short lived plasma cells after rituximab therapy, which in most models are associated with autoAbs. After treatment when repopulation of B cells occurs, there is a decreased proportion of memory B cells and an increase in transitional and naive B cells. [27] Accordingly, patients treated with rituximab are more likely to achieve long-term, clinical and immunological remission versus patients treated with corticosteroids. [29]

This study was designed to retrospectively evaluate autoAbs against TPO, DSG1, DSG3 and both muscarinic receptor 3 (M3) and nicotinic alpha (n) AChR in patients with diagnosed pemphigus vulgaris (PV) or pemphigus foliaceus (PF) and to correlate anti-TPO and anti-AChR titer levels with anti-DSG Abs, disease course, and medication regimen. We also tested IgG Varicella Zoster Virus (VZV) Ab levels at both time points to evaluate changes in Abs directed against foreign antigens in contrast to autoAbs. We compared results of patients on standard therapy of steroid sparing agents (SSA) versus those treated with rituximab, as SSAs control disease with less impact on autoAb levels. We hypothesize that levels of IgG autoAbs against TPO and/or AChRs from patients with pemphigus will not be correlated with clinical disease activity or levels of IgG autoAbs against DSG1 or DSG3. We also hypothesize that the changes in levels of IgG autoAbs against TPO and AChR will display a different response pattern after therapy (rituximab versus SSA) than seen with IgG anti-DSG1 and -DSG3.

## Material and methods

### Participants

Patients with PV or PF were chosen for this case-controlled, retrospective study from those seen in an autoimmune skin disease clinic at Duke University Medical Center. The patients chosen were previously consented to research studies that required the collection and storage of sera, and their consent forms specified that sera would be used to test for various Abs. This study was approved by the Institutional Research Board (Duke University, IRB #00085686). Normal controls (NC), defined as patients with no active skin disease, were chosen by random from the same sera bank and were also previously consented for prior research studies for future testing of sera.

### Inclusion criteria

Patients with either pemphigus vulgaris or foliaceus meeting both clinical and immunologic criteria (direct immunofluorescence proven keratinocyte cell surface IgG deposits in the epidermis) upon presentation were chosen for the study. Since desmoglein Ab titers have been shown to change with disease activity, patient sera for time point 1 were selected from patients with elevated DSG1 or DSG3 Ab titer level, a wide range of clinical disease activity, and who had follow-up appointments with subsequent sera collection and clinical disease assessment (S1 Table) (30).

### Exclusion criteria

Patients who were treated with IVIG or biologic agents, other than rituximab, within 1 year of sera collection were excluded.

### Clinical assessment of disease severity

Disease activity was retrospectively determined by review of medical record and measured using the Pemphigus Disease Area Index (PDAI). [30]

### Sample collection and testing

Approximately 10 mL of blood was collected from patients in serum-separating tubes. Blood samples were allowed to clot then centrifuged to 2500 G for 10 minutes at 4°C. Supernatant of sera was collected, labeled, and stored in -80°C freezers. Enzyme-linked immunosorbent assays (ELISA) were performed for IgG Abs against DSG1 and DSG3 (MBL Bion, Woburn, MA, USA), TPO and VZV (CalBioTech, El Cajon, CA, USA), M3-AChR (CellTrend GmbH, Luckenwalde, Germany) and n-AChR (MyBioSource, San Diego, CA, USA).

For anti -DSG1 and -DSG3 titers, samples were initially diluted 1:101. Values higher than the positive control were then further diluted until titers were within range per kit instructions. Values greater than 20 U/mL were considered positive for both anti-DSG1 and anti-DSG3. For anti-TPO and anti-VZV titers, samples were diluted 1:21, and Ab indices (ABI) were calculated based on the calibrator factor provided by the manufacturer for each kit. No sensitivity or normal ranges were described by the manufacturer for these assays. Samples were diluted 1:100 and 1:2 for anti-M3AChR and anti-nAChR ELISAs, respectively, and Ab concentration was determined using a standard supplied by the manufacturer. For the anti-M3AChR assay, normal values were reported as units/mL and the manufacturer described normal values as less than 6 units/ml. No sensitivity levels were reported. For the anti-nAChR test, sensitivity of the assay was reported as 1 ng/mL, and no normal range was reported by the manufacturer. In order to assess the role of dilution in the anti-M3AChR assay, selected sera were tested at a 1:20 dilution and compared to values obtained from normal sera tested at 1:20 dilution.

### Statistical analysis

Descriptive statistics were used to quantitatively describe and summarize findings. Due to the sample size, we analyzed the distribution and normality of data using the Shapiro-Wilk test. The data was determined to be asymmetric, and thus non-parametric 2-tailed analysis was performed with post-hoc Bonferroni correction for multiple comparisons. Ab levels were compared between disease groups (PF and PV) and NC using the Wilcoxon-Mann-Whitney rank sum test. The Bonferroni correction for multiple comparisons was utilized, which resulted in p < .0083 being utilized to determine statistical significance. Statistical analysis was done using software (Analyse-it Software, Ltd. https://analyse-it.com/).

## Results

### Patient characteristics

Our study cohort (n = 43) included 29 (67.4%) PV patients and 14 (32.6%) PF patients, as well as 20 NCs. Within the pemphigus cohort there were 22 males (51.2%) and 21 females (48.8%) with a male to female ratio of 1:0.95. The mean age (± SD) for all patients (PF and PV) was 52.6 (±13.8) years with a range of 20.2 to 76.6 years. The mean age for PV patients was 53.9 (±12.2) years with a range of 33.8 to 74.5 years, and the mean age for PF patients was 50.0 (±16.9) years with a range of 20.2 to 76.6 years. Average age of NC group was 36.8 (±10.1) years with a range from 22.1 to 57.4 years. (4 of the NC subjects did not have demographic data available). Treatment groups consisted of 21 patients treated with rituximab (14 PV, 7 PF) and 22 patients treated with SSAs (15 PV, 7 PF) (Table 1). The median time between T1 and T2 for PV patients was 727 days (PV SSA 970 days, PV rituximab 718 days) and for PF patients was 423 days (PF SSA 259 days, PF rituximab 464 days). The median disease duration at T1 for PV patients was 1.72 years (range: 0.05–30.8 years). For PF patients the median disease duration at T1 was 2.96 years (range: .35–8.4 years).

**Table 1:** Study participant demographics divided by treatment group and disease (PV and PF).

	NC*	All Patients	PV			PF		
			All	SSA	Rituximab	All	SSA	Rituximab
n	20	43	29	15	14	14	7	7
Male (%)	10	22 (51.2)	14 (48.3)	7 (46.7)	7 (50)	8 (57.1)	3 (42.9)	5 (71.4)
Female (%)	6	21 (48.8)	15 (51.7)	8 (53.3)	7 (50)	6 (42.9)	4 (57.1)	2 (29.6)
Avg Age (SD)	36.8 (10.1)	52.6 (13.8)	53.9 (12.2)	52.8 (11.2)	55.2 (13.5)	50.0 (16.9)	55.4 (18.5)	44.9 (14.3)

* Demographic data for 4 NCs was not available.NC, normal control; PF, pemphigus foliaceus; PV, pemphigus vulgaris; SSA, steroid sparing agent

### Disease severity

The average PDAI score at T1 for all patients was 20.0 (±19.4) with a range of 0 to 94. At T2, the average PDAI score for all patients was 2.0 (±3.9) with a range of 0 to 22, documenting significant clinical improvement during treatment (p<0.0001). At T1, the mean PDAI for patients with PV was 20.8 (±17.4) with a range of 0 to 61, and for PF subjects it was 18.4 (±23.5) with a range of 0 to 94. For patients with PV the mean PDAI at T2 was 2.2 (±4.5) with a range of 0 to 22 (p<0.0001) and for PF subjects was 1.6 (±2.1) with a range of 0 to 6 (p<0.0001). The breakdown of PDAI scores per treatment group can be found in Table 2.

**Table 2:** a (pemphigus vulgaris) and b (pemphigus foliaceus). Average (SD, Range) PDAI and antibody concentrations grouped by treatment and disease.

a					
	NC	All Patients	PV		
			All	SSA	Rituximab
PDAI T1	-	**20** (19.4, 0–94)	**20.8** (17.4, 0–61)	**13** (17, 0–61)	**28.9** (14, 3–61)
PDAI T2	-	**2.0** (3.9, 0–22)	**2.2** (4.5, 0–22)	**2.3** (5.7, 0–22)	**2.1** (3, 0–9)
DSG1 T1	**1.45** (3.5, 0–16)	**544.4** (1168, 0–5731)	**221.7** (838, 0–4562)	**333.8** (1170, 0–4562)	**101.6** (103, 0–325)
DSG1 T2	-	**39.4** (87, 0–490)	**6.7** (16.8, 0–89)	**8** (22.6, 0–89)	**5.2** (7.2, 0–24)
DSG3 T1	**0.65** (1.1, 0–5)	**980** (1683, 0–8841)	**1450** (1883, 81–8841)	**1656** (2159, 82–8841)	**1230** (1586, 112–5153)
DSG3 T2	-	**264** (524, 0–2076)	**391** (600, 1–2076)	**552** (617, 11–2076)	**218** (551, 1–2076)
nAChR T1	**18.2** (3.1, 12.8–25.4)	**28.2** (14, 9–97)	**21.8** (5.2, 9–31)	**20.9** (5.9, 9.3–30.8)	**22.8** (4.2, 16–30)
nAChR T2	-	**27.7** (15.1, 12–104)	**20.8** (5.5, 12–38)	**21.5** (6.1, 14–38)	**19.9** (4.9, 12–29)
m3AChR T1	**3.7** (1.6, 1.9–8.6)	**3.2** (1.8, 0.8–9.6)	**3.5** (1.9, 1.6–9.6)	**3.2** (1.3, 1.6–5.8)	**3.9** (2.4, 1.8–9.6)
m3AChR T2	-	**3.2** (1.9, 0.5–11.4)	**3.4** (2, 1.7–11.4)	**3.9** (2.5, 1.7–11.4)	**2.9** (1.2, 1.8–6.1)
TPO T1	**0.29** (0.35, 0.06–1.5)	**0.21** (0.36, 0.04–1.83)	**0.23** (0.35, .04–1.83)	**0.32** (0.47, 0.05–1.83)	**0.126** (0.09, 0.04–0.4)
TPO T2	-	**0.17** (0.17, 0.03–0.74)	**0.18** (0.17, 0.03–0.74)	**0.26** (0.22, 0.06–0.74)	**0.1** (0.03, 0.03–0.19)
VZV T1	**2.84** (0.82, 1.3–4.0)	**2.40** (1.15, 0.12–5.5)	**2.36** (0.98, 0.12–4.1)	**2.58** (1.15, 0.12–4.12)	**2.12** (0.73, 0.69–3.36)
VZV T2	-	**2.69** (1.24, 0.11–6.1)	**2.73** (1.0, 0.12–4.47)	**2.97** (1.13, 0.12–4.5)	**2.49** (0.83, 0.94–3.9)
b					
	NC	All Patients	PF		
			All	SSA	Rituximab
PDAI T1	-	**20** (19.4, 0–94)	**18.4** (23.5, 0–94)	**14** (10.5, 0–33)	**22.7** (32, 0–94)
PDAI T2	-	**2.0** (3.9, 0–22)	**1.6** (2.1, 0–6)	**2.4** (2.6, 0–6)	**0.7** (1.2, 0–3)
DSG1 T1	**1.45** (3.5, 0–16)	**544.4** (1168, 0–5731)	**1212** (1476, 130–5731)	**933** (750, 211–2376)	**1492** (1995, 131–5731)
DSG1 T2	-	**39.4** (87, 0–490)	**107** (129, 1–490)	**134** (166, 1–490)	**80.4** (84, 1–219)
DSG3 T1	**0.65** (1.1, 0–5)	**980** (1683, 0–8841)	**6.8** (11, 0–39)	**3.4** (55, 0–14)	**10** (14, 0–39)
DSG3 T2	-	**264** (524, 0–2076)	**0.67** (0.73, 0–2.7)	**0.6** (0.44, 0.08–1.1)	**0.74** (0.96, 0–2.7)
nAChR T1	**18.2** (3.1, 12.8–25.4)	**28.2**(14, 9–97)	**41.6** (17.2, 21–97)	**46.8** (22.3, 31–97)	**36.4** (9, 21–47)
nAChR T2	-	**27.7** (15.1, 12–104)	**42.1** (18.6, 29–104)	**46** (25, 35–104)	**37.9** (7.9, 28.6–49.2)
m3AChR T1	**3.7** (1.6, 1.9–8.6)	**3.2** (1.8, 0.8–9.6)	**2.5** (1.3, 0.8–6.1)	**2.3** (1.1, 0.8–4)	**2.7** (1.6, 1.4–6.1)
m3AChR T2	-	**3.2** (1.9, 0.5–11.4)	**2.7** (1.4, 0.5–6.82)	**2.8** (0.73, 1.8–3.9(	**2.7** (2, 0.5–6.8)
TPO T1	**0.29** (0.35, 0.06–1.5)	**0.21** (0.36, 0.04–1.83)	**0.18** (0.32, 0.04–1.25)	**0.103** (0.06, 0.05–0.24)	**0.256** (0.44, 0.075–1.25)
TPO T2	-	**0.17** (0.17, 0.03–0.74)	**0.14** (0.18, 0.05–0.73)	**0.11** (0.07, 0.05–0.27)	**0.18** (0.25, 0.06–0.73)
VZV T1	**2.84** (0.82, 1.3–4.0)	**2.40** (1.15, 0.12–5.5)	**2.5** (1.5, 0.4–5.5)	**2.45** (1.72, 0.91–5.5)	**2.55** (13, 0.41–4.8)
VZV T2	-	**2.69** (1.24, 0.11–6.1)	**2.59** (1.7, 0.62–6.1)	**2.36** (1.8, 0.94–6.09)	**2.82** (1.6, 0.6–5.7)

DSG1, desmoglein 1 (U/mL); DSG3, desmoglein 3 (U/mL); m3AChR, muscarinic 3 Acetylcholine Receptor (U/mL); NC, normal control; PDAI, Pemphigus Disease Area Index; PF, pemphigus foliaceus; PV, pemphigus vulgaris; T1, timepoint 1; T2, timepoint 2; nAChR, nicotinic Acetylcholine Receptor (ng/mL); SSA, steroid sparing agent; TPO, thyroid peroxidase (antibody binding index, ABI); VZV, Varicella Zoster Virus (ABI)

The distribution of the PDAI scores at T1 and T2 as related to clinical disease status, as described by Boulard et al, are shown in S1 Table. [31] Using the categorization of no disease activity (PDAI = 0), moderate disease activity (PDAI 1–15), significant disease activity (PDAI 16–45) or extensive disease activity (PDAI > 45) reveals that 13 of 29 (45%) PV subjects had no or moderate disease activity at T1 whereas 10 of 14 (71%) PF subjects had no or moderate disease activity at T1. At T2, 28 of 29 (97%) of PV patients and all PF patients had no or moderate activity.

### Antibody levels

**Fig 1:**
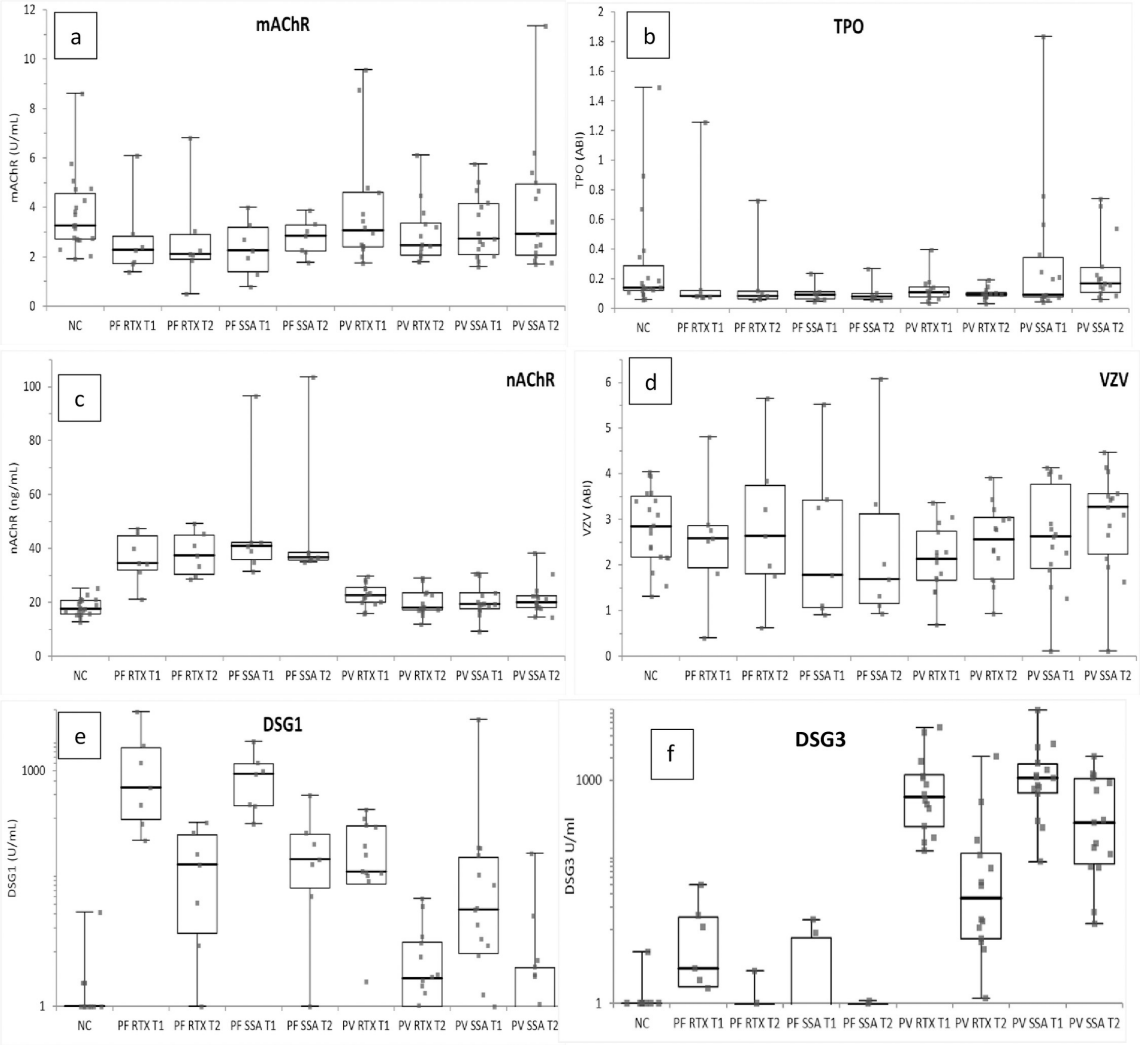
Scatter plots with range (whiskers), upper and lower quartiles (boxes), and medians (horizontal lines) of all tested Abs. Comparing T1 to T2, there was no significant difference in M3AChR (1a), TPO (1b), nAChR (1c), or VZV (1d) Abs. There was a significant decrease in anti- DSG1 (1e) and–DSG3 (1f) Ab levels from T1 to T2 (PV DSG1 SSA p = .00025, RTX p = .0025; PV DSG3 RTX p = .00001; PF DSG1 SSA p = .0041, RTX p = .0041). Wilcoxon-Mann-Whitney was used, and after Bonferroni correction, p < .0083 was determined to be statistically significant. PF, pemphigus foliaceus; PV, pemphigus vulgaris; SSA, steroid sparing agent; RTX, rituximab.

Analysis of the anti-DSG Ab levels for patients with PV revealed elevation of the mean IgG anti-DSG1 levels at T1 (221.7 U/mL with a range from 0 to 4562 U/mL) and anti-DSG3 (1450 U/mL with a range from 81 to 8841 U/mL). These values were significantly increased compared to normal subjects (p<0.0001, Table 2, Fig 1). In patients with PF, mean IgG anti-DSG1 levels were significantly higher at T1 than those seen in NC (PF = 1212 U/mL, range 131–5731 U/mL; NC = 1.45 U/mL, range 0–16 U/mL; p<0.001).

In contrast, the T1 levels of anti-TPO Abs (0.21 ABI with a range from 0.04 to 1.83 ABI), anti-m3AChR Abs (3.2 U/mL with a range from 0.8 to 9.6 U/mL) and anti-VZV Abs (2.40 ABI with a range from 0.12 to 5.5 ABI), were not significantly different in pemphigus patients from the normal controls. As the testing for anti-M3AChR Abs was conducted at a serum dilution of 1:100, we also studied PV and normal sera at a dilution of 1:20. No difference was noted in the levels of anti–M3AChR Abs between the normal population and the PV sera tested with either dilution of sera. There was, however, the expected increase in absolute amount of anti- M3AchR Ab detected in the 1:20 diluted samples from both the normal subjects and pemphigus patients, but there was no significant difference between the normal subjects and patients. (S1A and S1B Fig). Anti-nAChR Abs (28.2 ng/mL with a range from 9–97 ng/mL) however were significantly higher in pemphigus patients than that seen for NC (18.2 ng/ml with a range from 12.8–25.4 ng/mL, p < .0001). Analysis of pemphigus subtypes showed that in PV, the T1 levels of anti-nAChR Abs were significantly different from that seen in normal controls, however no significant difference could be seen in any other Abs (Table 2). In contrast, in patients with PF, IgG anti-nAChR were significantly different than that seen in normal subjects (p<0.0001). Of interest, in the PF patients, IgG anti-M3AChR were lower than seen in NC (PF = 2.5 U/mL; NC = 3.7 U/mL; p = 0.0075) while anti-TPO and anti-VZV Abs were not significantly different from NC subjects (Table 2).

In order to determine a potential role for these Abs in the pathogenesis of the disease, we analyzed Ab levels at T2, when clinical disease was significantly improved as confirmed by significantly decreased PDAIs. At T2, the mean anti-DSG1 Ab titer for all PV patients was 6.7 U/mL with a range from 0 to 89 U/ml. At T2, the mean IgG anti-DSG3 Ab titer in PV patients was 391 U/mL with a range from 1 to 2076 U/ml. Both IgG anti-DSG1 and anti-DSG3 Abs were significantly lower at T2 when compared to the T1 levels (Wilcoxon-Mann-Whitney, p<0.00001) consistent with a significant decrease in the PDAI of the majority of patients. Significant reduction in anti-DSG1 Ab levels were seen in PV patients regardless of therapy with SSA (p = 0.00023) or rituximab (p = 0.0025), whereas anti-DSG3 Abs while lower in both treatment groups were only statistically significant in rituximab treated PV patients (p = 0.00001, Fig 1). For patients with PF, a similar significant reduction in IgG anti-DSG1 levels was seen between T1 and T2 (Wilcoxon-Mann-Whitney, p < 0.0001). This finding was consistent with the improvement in clinical disease severity that was seen in the PDAI scores of the majority of patients with PF and was seen regardless of therapy with SSA or rituximab (Fig 1).

In order to determine if similar changes in non-desmoglein Ab levels occur with clinical disease improvement, we assessed the level of these Abs at time T2 and compared them to T1 Ab levels in pemphigus patients (PV and PF) and in each type of therapy group (SSA and rituximab). Although there was a significant decrease in the levels of anti-DSG Abs and PDAI in all patients with pemphigus between T1 and T2, no significant difference was seen when comparing IgG anti-TPO, anti-nAChR, anti-M3AChR or anti-VZV (Fig 1). Similarly, no difference was seen when comparing patients with PV or PF and across both treatments (SSA and rituximab). These findings demonstrate that there were no alterations in non-desmoglein autoAb levels in our patients with pemphigus despite clear demonstration of a clinical response and significant decrease in anti-DSG Abs.

## Discussion

In this paper, we describe 43 pemphigus patients with either PV or PF, diagnosed by clinical presentation as well as direct immunofluorescence proven cell surface IgG deposits in the epidermis. These patients were either treated with 1) prednisone and SSA or 2) prednisone and rituximab (Table 3). The mean age, gender distribution, as well as disease activity as determined by retrospective PDAI were similar between both disease groups (PV and PF) as well as between both treatment groups (SSA and rituximab) (Table 1). A significant decrease in disease activity from T1 to T2, as evidenced by a decrease in PDAI score as well as a decrease in anti-DSG1 and -DSG3 Ab titers, was seen in both PF and PV patients and was seen in both treatment groups (Fig 1E and 1F, Table 2, S1 Table).

**Table 3:** Patient treatment and medications at T1 and T2.

Patient #	PV vs PF	Treatment Group	Prednisone (mg/day) T1	Adjuvant Rx (mg/day) T1	Prednisone (mg/day) T2	Adjuvant Rx (mg/day) T2	Time from rituximab to T2 (months)
1	PF	Rituximab	0	MMF 3000	0	MMF 1500	13
2	PF	SSA	5	MMF 1500	0	Dapsone 100	n/a
3	PF	SSA	0	Dapsone 100 q3 days	0	-	n/a
4	PF	Rituximab	60	-	0	-	13
5	PF	Rituximab	20	-	0	-	4
6	PF	SSA	60	-	5	AZ 100	n/a
7	PF	Rituximab	0	AZ 350	0	AZ 50	12
8	PF	Rituximab	0	MMF 1500	0	MMF 1500	12
9	PF	SSA	60	-	10	-	n/a
10	PF	Rituximab	60	MMF 2000	0	-	8
11	PF	SSA	0	HCQ 200	0	HCQ 200	n/a
12	PF	SSA	40	-	0	AZ 100	n/a
13	PF	Rituximab	20	-	0	-	15
14	PF	SSA	0	AZ 100	0	AZ 100	n/a
15	PV	SSA	80	-	0	-	n/a
16	PV	SSA	2.5	-	0	AZ 200	n/a
17	PV	Rituximab	0	-	0	-	10
18	PV	SSA	60	-	0	AZ 50	n/a
19	PV	SSA	0	-	0	AZ 150	n/a
20	PV	Rituximab	60	MMF 2000; AZ 200	0	-	14
21	PV	Rituximab	80	-	0	-	12
22	PV	Rituximab	0	-	0	-	10
23	PV	SSA	5	MMF 2500	10	-	n/a
24	PV	SSA	10	Imuran 100	10	-	n/a
25	PV	Rituximab	20	-	0	-	14
26	PV	SSA	0	AZ 150	0	AZ 200	n/a
27	PV	Rituximab	0	Imuran 150	5	-	10
28	PV	SSA	60	Imuran 150	7	AZ 150	n/a
29	PV	Rituximab	0	-	15	MMF 2000	12
30	PV	SSA	50	-	10	AZ 100	n/a
31	PV	Rituximab	60	-	0	-	12
32	PV	SSA	10	CP 75	2.5	CP 50	n/a
33	PV	SSA	30	MTX 7.5 (mg/wk)	50	MMF 2000	n/a
34	PV	Rituximab	30	AZ 150; NAA 1000	0	AZ 50	13
35	PV	SSA	0	Doxy 200	30	MMF 2000	n/a
36	PV	Rituximab	20	MMF 1000	0	MMF 1000	11
37	PV	SSA	20	MMF 1000	5	MMF 1500	n/a
38	PV	Rituximab	3	-	10	-	2
39	PV	Rituximab	60	-	0	-	4
40	PV	SSA	40	-	0	MMF 2000	n/a
41	PV	Rituximab	0	MMF 2500	20	MMF 3000	4
42	PV	SSA	40	-	10	MMF 2000	n/a
43	PV	Rituximab	60	-	5	-	3

For rituximab treatment group, time between infusion and T2 is provided. If patient was not on adjuvant therapy a (-) is present. All medications are listed in mg/day unless otherwise noted. AZ, azathioprine; CP, cyclophosphamide; Doxy, doxycycline; HCQ, hydroxychloroquine; MTX, methotrexate; MMF, mycophenolate mofetil; NAA, niacinamide; PF, pemphigus foliaceus; PV, pemphigus vulgaris; SSA, steroid sparing agent.

At T1, there was no difference in the levels of VZV, TPO or M3AChR Abs in patients with pemphigus compared to NCs. There was, however, a higher level of the nAChR Abs in pemphigus patients compared to NC. This was noted in patients with PV at T1 only and at both T1 and T2 in PF patients. There was also lower anti-M3AChR (T1 only) in PF patients when compared to normal controls. This difference was not seen in the PV patients or in the total pemphigus patient population.

In order to assess if these non-DSG autoAbs may play a role in the clinical disease of pemphigus patients, we compared changes in all Ab levels from T1 to T2 along with assessing changes in disease activity. Comparing T1 to T2, there was no difference in M3AChR, nAChR or TPO autoAbs or in VZV Ab levels. There was, however, a significant decrease in the anti- DSG1 and -DSG3 Ab levels as well as in clinical disease activity, as measured by the PDAI, for both PV and PF patients. It is important to note, however, that levels of both anti-DSG1 and–DSG3 remained abnormal in many subjects with both PV and PF (Fig 1, Table 2). These data demonstrate that out of these suspected autoAbs, only nAChR showed a higher concentration in our patients with pemphigus when compared to normal controls at a time when disease activity was higher (T1) for PV patients and at both timepoints for PF patients. Of importance, however, there was no significant change in Ab concentration in these non-DSG autoAbs (both PV and PF patients or with both SSA and rituximab treatment groups) even though there was clinical improvement in our pemphigus patients (Fig 1, Table 2, S1 Table). This suggests that these non–DSG autoAbs are not playing a critical role in the clinical manifestations of pemphigus in our patient population. As a control, we also analyzed the levels of a non-autoAb, IgG anti-VZV in our pemphigus patients. No difference was noted in VZV Ab levels when compared to normal subjects or between T2 from T1.

Previous studies have demonstrated that patients with pemphigus have elevated levels of autoAbs directed at muscarinic acetylcholine receptors (M3–5) and anti-TPO Abs. [12, 17, 20, 25] The development of new techniques for measuring Abs has resulted in the identification of a large number of autoantigens with detectable autoAbs in the sera of patients with pemphigus and other autoimmune diseases. [32] Although numerous in vitro and in vivo animal studies have demonstrated that disruption of acetylcholine receptor function (nicotinic and muscarinic AChR) results in acantholysis similar to that seen in pemphigus, the exact role of these autoAbs in the clinical manifestations of pemphigus in man is unclear. [33] Recently, Lakshmi and co-workers reported that patients with PV and PF had significantly higher titers of IgG anti-M3AChR than normal controls, as well as Abs against DSG1 and DSG3. [20] Furthermore, they reported that the level of anti-M3AChR autoAbs correlated with clinical disease activity as determined by PDAI as well as with anti-DSG1 and anti-DSG3 Ab levels and that all of the autoAbs declined with clinical improvement.

Using conventional ELISA assays, we set out to determine if our patients with pemphigus had Abs against three of the dominant non-DSG autoantigens, nAChR, M3AChR, and TPO, and if those Ab levels correlated with the levels of anti-DSG Abs, clinical disease activity, and response to therapy. We also sought to compare any changes that occur in the autoAb levels with the type of therapy with either prednisone plus steroid sparing agents or prednisone plus rituximab.

Except for nAChR Ab levels, we did not find any significant elevation of the non-DSG autoAbs in our patients when compared to normal controls. Moreover, we did not see any change in the levels of non-DSG autoAbs during therapy and after disease control. These results are different than the data presented in other papers that looked at similar non-desmoglein autoAbs, however there may be several reasons for the differences in results that are seen. [20–24]

Differences in patient populations may have contributed to the results that we found in this study versus what has previously been published. Comparison of the general demographics of patient populations revealed no significant differences in age, gender, or baseline disease severity from previous studies (S2 Table). [13, 17, 20] Our patients, however, were mostly from the southeast United States. Many studies have evaluated the differences in genetics, prevalence of pemphigus (vulgaris versus foliaceus versus others), and gender distributions in populations across the world. [34] The polygenic foundation that includes multiple genetic foci has been established for both PV and PF and may contribute to diverse autoAbs developing in different pemphigus populations. [17] Lakshmi and co-workers, who reported a correlation of MAChR Ab with disease activity, studied an Indian patient population. Our pemphigus patients were predominantly Caucasian with only 4 subjects of Indian descent, preventing an effective subgroup analysis. [20] Additionally, it is known that specific HLA associations are related to the expression of non-DSG Abs. We did not do genetic testing on our patients for this study and cannot comment on their HLA genetic makeup. [17, 25]

Another potential variable that may play a role in different autoAb profiles includes geographical regions where the patient develops pemphigus. Different environmental antigens may trigger the autoimmune response which may also influence the variations seen in our results versus other studies. Several studies have linked exposure to an environmental antigen with pathogenic autoAbs in patients with pemphigus, and it would be of interest to determine if the presence of non-DSG autoAbs is different in these populations. [35–38] Additionally, many different drugs have been found to incite the development of pemphigus as well as viral infections and physical agents. [16, 39–42] It is possible that the different initiating events in patients with pemphigus play a key role in the development of the autoAb profile. There have not been studies looking at the changes in autoAb levels (DSG1, DSG3, TPO, AChR) in patients separated by the inciting event that triggered the onset of disease. It is highly plausible that patients in different parts of the world would be exposed to some of these triggers more often than others.

As the theory that non-desmoglein Abs may be involved in pemphigus has gained interest, many recent studies have tried to elucidate their role and prevalence in disease populations. [17, 19, 20] These studies, however, utilize many different assays and techniques with varying sensitivity. Our study utilized enzyme-linked immunosorbent assays (ELISA) to test for concentration of Abs, similar to that utilized by Lakshmi and co-workers. [20] Many of the studies that have found increases in anti- TPO and -AChR Abs, as well as Abs against other autoantigens, utilized immunoblotting, immunofluorescence, PCR, western blots, proteomics, and multiplexed microarrays. [15, 17, 19, 25, 43] Studies comparing the sensitivity of microarray ELISAs vs conventional ELISAs have shown both similar sensitivities and an increase sensitivity of microarray techniques. [44, 45] It would be of interest to exchange sera with other laboratories to determine what the role of assay differences may play in these differences.

Although we did not find significant levels of non-DSG autoAbs in our patients with pemphigus, it is also possible that these Abs may occur at variable times in the disease cycle. It is conceivable that the non-DSG autoAbs may be either an early or a late phenomenon in the course of disease. Although we did not see any difference in results related to the type of treatment, it is not possible to conclude that the therapy of these patients did not influence our results. Additionally, it is possible that the number of subjects in our study was insufficient to detect a specific clinical or immunologic subset of patients for whom non-DSG auto antibodies play a critical role in their pathogenesis. Further studies will be needed to assess this possibility.

Finally, Seiffert-Sinha and co–workers have reported an increased frequency of anti-TPO Abs that was highest in those subjects who had active disease and did not have elevated levels of IgG anti- DSG1 or DSG3 Abs. [25] We did not have any patients with PV lacking both IgG anti- DSG1 and -DSG3 Abs, and none of our subjects with PV with IgG anti-DSG3 alone had levels of IgG anti-TPO outside of the normal range.

In any case, our study demonstrates that there is heterogeneity in the expression of non–DSG autoAbs in different pemphigus populations. These differences may be a result of differences in the patient populations (HLA or other genetic differences), different initiating events, different stages of disease or other factors. The strong correlation of anti-DSG autoAb levels with clinical disease activity in the majority of our patients and by other studies provides further evidence of the key role of anti-DSG Abs in the pathogenesis of the clinical disease. The presence, however, of non-DSG autoAbs in many different studies and different patient populations suggest that continued study of these Abs will provide important information related to autoimmunity in general, to potential unique clinical and or immunological subsets of pemphigus patients and to the etiology and pathogenesis of pemphigus.

## Conclusions

We believe that the minimal variance from normal controls and pemphigus patients in the levels of non-DSG autoAbs, coupled with the lack of variation with treatment and the lack of correlation with IgG anti- DSG1 and -DSG3 autoAb levels and clinical improvement suggests that these Abs may not play a direct role in the pathogenesis of the disease in our patient population. Differences in the genetic backgrounds, initiating events, disease duration and/or treatments all may play a critical role in the development of rare distinct clinical subtypes and result in the variable results that are seen in our study. Further studies including the exchange of sera are needed to address these issues.

## Supporting information

S1 Fig Box and Whiskers plot of IgG anti-M3AChR antibody levels in 11 patients with PV and 11 normal subjects tested at 1:20 dilutions (1A) and 1:100 dilutions (1B). No significant increase noted in the level of IgG anti-M3AChR antibody between normal subjects and patients with PV with either dilution. Increased serum concentration did not alter the distribution of serum values between normal and PV subjects.
(DOCX)

S1 Table (DOCX)

S2 Table (DOCX)
