# Supplementary material for: Correlation of IgG autoantibodies against acetylcholine receptors and desmogleins in patients with pemphigus treated with steroid sparing agents or rituximab
Source: PLoS One. 2020 Jun 18;15(6):e0233957. doi: 10.1371/journal.pone.0233957 (PMC7302486; doi:10.1371/journal.pone.0233957)
Supplement: S1 Fig — No significant increase noted in the level of IgG anti-M3AChR antibody between normal subjects and patients with PV with either dilution. Increased serum concentration did not alter the distribution of serum values between normal and PV subjects. (DOCX) [file pone.0233957.s001.docx]

Figure 1As


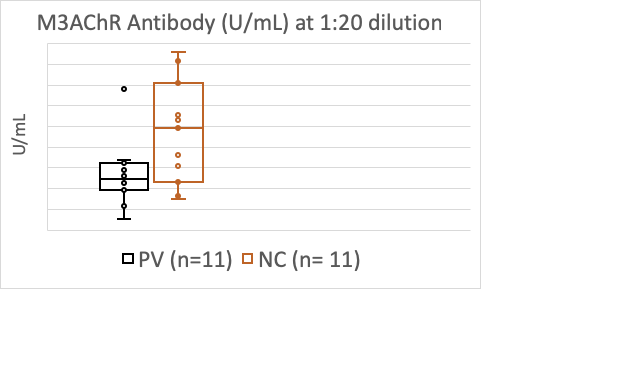


Figure 1Bs


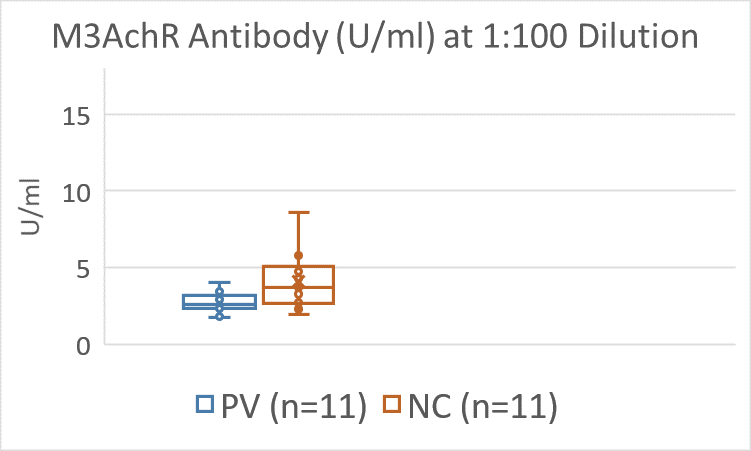


Figure 1A and 1Bs:

Box and Whiskers plot of IgG anti-M3AChR antibody levels in 11 patients with PV and 11 normal subjects tested at 1:20 dilutions (1A) and 1:100 dilutions (1B). No significant increase noted in the level of IgG anti-M3AChR antibody between normal subjects and patients with PV with either dilution. Increased serum concentration did not alter the distribution of serum values between normal and PV subjects.
