# Supplementary material for: Correlation of IgG autoantibodies against acetylcholine receptors and desmogleins in patients with pemphigus treated with steroid sparing agents or rituximab
Source: PLoS One. 2020 Jun 18;15(6):e0233957. doi: 10.1371/journal.pone.0233957 (PMC7302486; doi:10.1371/journal.pone.0233957)
Supplement: S1 Table — (DOCX) [file pone.0233957.s002.docx]

Table S1

| Clinical Disease Activity (PDAI score) | T1 PV | T2 PV | T1 PF | T2 PF |
| --- | --- | --- | --- | --- |
| No disease activity (0) | 2* | 17 | 2* | 8 |
| Moderate (1-15) | 11 | 11 | 8 | 6 |
| Significant (16 -45) | 13 | 1 | 3 | 0 |
| Extensive (>45) | 3 | 0 | 1 | 0 |

Number of PF and PV subjects with indicated disease activity at each time point. *Patients at end of consolidation phase at time T1. PDAI, Pemphigus Disease Area Index; PF, pemphigus foliaceous; PV, pemphigus vulgaris.
