# Supplementary material for: Correlation of IgG autoantibodies against acetylcholine receptors and desmogleins in patients with pemphigus treated with steroid sparing agents or rituximab
Source: PLoS One. 2020 Jun 18;15(6):e0233957. doi: 10.1371/journal.pone.0233957 (PMC7302486; doi:10.1371/journal.pone.0233957)
Supplement: S2 Table — (DOCX) [file pone.0233957.s003.docx]

Table S2

|  | Bhatia et al | Lakshmi et al (20) | Nguyen et al (12) | Sajda et al (17) |
| --- | --- | --- | --- | --- |
| Number Pemphigus Subjects | 43 | 45 | 40 | 40 |
| PV subjects | 29 | 35 | 34 | 39 |
| PF Subjects | 14 | 10 | 6 | 1 |
| Male/Female | 22/21 | 21/24 | 17/23 | 14/26 |
| Mean Age (Range) years | 52.6 (20-77) | 44.7 (7-75) | NR (31-89 0 | 53.2 (21-76) |
| Baseline PDAI PV (Mean) | 21 | 26 | NR | NR |
| Baseline PDAI PF (Mean) | 18 | 30.3 | NR | NR |
